# IFN-γ secreting capacity of CD8+T cell is compromised with the increased copies of epitope encoding sequences in DNA vaccine design

**DOI:** 10.1186/1742-4690-9-S2-P311

**Published:** 2012-09-13

**Authors:** J Wang, Y Wan, J Xu

**Affiliations:** 1 Fudan University, Shanghai, China; 2 Shanghai Public Health Clinical Center, Shanghai, China

## Background

Epitope based vaccines are widely used in vaccine development against HIV-1, cancer and other diseases. One of the key components is how to enhance its immunogenicity. Previous studies suggested that the magnitudes of both T cell and antibody responses could be improved through repeating epitope in design, however, it remains unknown how this could influence the functional features of T cell responses and its optimization.

## Methods

A previously indentified HIV-1_RL42_ envelope T cell epitope GIRKNYQHLWRWGTM (Env2) was employed, mini-genes encoding a single, triplicated or sextuplicated copies of this epitope were synthesized and inserted into a DNA vector. To enhance their expression efficiency and immunogenicity, Kozak sequence, ER signal sequence and an universal Th2 epitope were introduced, His tag was added to detect its expression. In vitro expression was confirmed by transfection and immunoprecipitation. C57B/C mice were inoculated i.m. and sacrificed to do in vivo assessment. ICS assays were used to read out Env2-specific immune responses. Statistical analysis was done with Prism5.0 software.

## Results

It’s showed that all three mini-gene DNA vaccines could elicited appreciable IFN-γresponses in CD8+ T cells, no significant IL-2 secretion was observed. One way ANOVA analysis showed that the frequencies of IFN-γ+CD8+T cells induced ranked as single copy of Env2< triplicated < sextuplicated (P=0.02). Further analysis indicated that MFI of IFN-γ+CD8+T cells decreased along with the increasing of epitope copy number, which was single-Env2 group>triplicated>sextuplicated (p=0.09). When a single copy Env2 and the combined data from triplicated- and sextuplicated-Env2 were compared, we observed MFI in single copy Env2>multiple copy (p=0.004).

## Conclusion

Our data confirmed previous observation that repeated epitope design could improve the frequencies of specific T cells. Interestingly, we demonstrated that the IFN-γ secreting capacity for individual T cell might be compromised along with the increased responding frequencies, which should be taken into consideration in vaccine design.

